# Association of ZWINT Expression with Clinicopathologic Characteristics and Prognosis in Breast Cancer Patients

**DOI:** 10.1007/s11596-025-00081-9

**Published:** 2025-07-01

**Authors:** Bei Liu, Qin Wang, Xiao-hong Min, Han-han Liu, Huan Wu, Hui Xu, Jun-bo Hu, Yong-qing Tong, Zi-ming Huang

**Affiliations:** 1 Department of Pathology, Maternal and Child Health Hospital of Hubei Province, Wuhan, 430070 China; 2 Clinical Molecular Diagnostic Center, Renmin Hospital of Wuhan University, Wuhan, 430060 China; 3 Department of Breast and Thyroid Surgery, Maternal and Child Health Hospital of Hubei Province, Wuhan, 430070 China

**Keywords:** ZW10 interacting kinetochore protein (ZWINT), Breast cancer, Prognosis, Public databases, Prognostic value, Comprehensive analysis

## Abstract

**Objective:**

ZW10 interacting kinetochore protein (ZWINT) has been demonstrated to play a pivotal role in the growth, invasion, and migration of cancers. Nevertheless, whether the expression levels of ZWINT are significantly correlated with clinicopathological characteristics and prognostic outcomes of patients with breast cancer remains elusive. This study systematically investigated the clinical significance of ZWINT expression in breast cancer through integrated molecular subtyping and survival analysis.

**Methods:**

We systematically characterized the spatial expression pattern of ZWINT across various breast cancer subtypes and assessed its prognostic significance using an integrated bioinformatics approach that involved multi-omics analysis. The approach included the Breast Cancer Gene-Expression Miner v5.1 (bc-GenExMiner v5.1), TNMplot, MuTarget, PrognoScan database, and Database for Annotation, Visualization, and Integrated Discovery (DAVID).

**Results:**

Our analysis revealed consistent upregulation of ZWINT mRNA and protein expression across distinct clinicopathological subtypes of breast cancer. ZWINT overexpression demonstrated significant co-occurrence with truncating mutations in cadherin 1 (CDH1) and tumor protein p53 (TP53), suggesting potential functional crosstalk in tumor progression pathways. The overexpression of ZWINT correlated with adverse clinical outcomes, showing 48% increased mortality risk (overall survival: HR 1.48, 95% CI 1.23–1.79), 66% higher recurrence probability (relapse-free survival: 1.66, 95% CI 1.50–1.84), and 63% elevated metastasis risk (distant metastasis-free survival: HR 1.63, 95% CI 1.39–1.90). Multivariate Cox regression incorporating TNM staging and molecular subtypes confirmed ZWINT as an independent prognostic determinant (*P* < 0.001, Harrell’s C-index = 0.7827), which was validated through bootstrap resampling (1000 iterations).

**Conclusion:**

ZWINT may serve as a potential biomarker for prognosis and a possible therapeutic target alongside TP53/CDH1 in breast cancer.

**Supplementary Information:**

The online version contains supplementary material available at 10.1007/s11596-025-00081-9.

## Introduction

Breast cancer is the most common tumor in women, accounting for 31% of female malignant tumors. Its incidence rate is increasing at the rate of 0.5% per year [1]. Recently, the treatment of breast cancer has greatly changed according to its molecular typing. For estrogen receptor (ER)-positive and/or progesterone receptor (PR)-positive tumors, hormone therapy is the main treatment, with chemotherapy incorporated according to tumor size, grade, and lymph node status. For human epidermal growth factor receptor 2 (HER2)-positive tumors, anti-HER2 targeted therapy combined with chemotherapy is the cornerstone of treatment. In contrast, triple-negative breast cancers (TNBCs), which lack the expression of ER, PR, and HER2, rely on chemotherapy as their main treatment modality [2]. Therefore, ER, PR, and HER2 are important predictive markers for breast cancer. In addition, more and more proteins such as Ki67, serine/threonine kinase 15 (STK15), and survivin are also included in prognostic biomarkers to predict breast cancer behavior and/or response to treatment, and to better understand the prognosis and treatment of the disease [3].

ZW10 interacting kinetochore protein (ZWINT) is a recognized element of the kinetochore complex, essential for the mitotic spindle checkpoint and for maintaining the mitotic cycle [4]. The kinetochore is a sophisticated structure that plays a pivotal role in various critical processes during cell division. This tri-laminar plate, to which microtubules are anchored, links chromosomes to the spindle apparatus, ensuring the precise segregation of chromosomes during both mitosis and meiosis [4]. Abnormalities in mitosis are frequently observed in many cancers. While the specific roles of the kinetochore’s molecular components and their interactions remain largely undefined, increasing evidence indicates that ZWINT is often overexpressed in various human cancers, correlating with unfavorable clinical outcomes and early recurrence [5–7]. However, the precise biological functions and clinical implications of ZWINT in breast cancer remain uncertain. Limited research has been conducted to determine whether it can serve as a prognostic biomarker for the molecular classification of breast cancer.

In this work, we comprehensively analyzed the data from multiple web-based genomic databases and observed consistently elevated expression of ZWINT mRNA and protein in breast cancer with various clinicopathological characteristics. Our analysis further revealed significant associations between ZWINT expression and mutations in the key tumor suppressor genes, particularly truncating mutations in cadherin 1 (CDH1) and tumor protein p53 (TP53). Importantly, elevated ZWINT mRNA expression demonstrated strong correlations with poorer clinical outcomes, including reduced overall survival (OS), relapse-free survival (RFS), distant metastasis-free survival (DMFS), and post-progression survival (PPS). Additionally, ZWINT was found to be a useful independent prognostic marker for breast cancer using Cox regression analysis.

## Materials and Methods

### TNMplot Database Analysis

The TNMplot tool (https://www.tnmplot.com/) is used to analyze the differential gene expression in tumor tissues, normal tissues, and metastatic tissues. TNMplot includes 56,938 unique multilevel quality-controlled samples from the gene expression omnibus (GEO), genotype-tissue expression (GTex), the cancer genome atlas (TCGA), and the therapeutically applicable research to generate effective treatments (TARGET) databases, along with 15,648 normal, 40,442 tumor, and 848 metastatic samples. With the use of this tool, the expression of ZWINT in normal, malignant, and metastatic tissues was compared and examined.

### Gene Expression Profiling Interactive Analysis (GEPIA)

The GEPIA tool (http://gepia.cancer-pku.cn/) is used to analyze the differential gene expression in tumor tissues and normal tissues. GEPIA includes the RNA sequencing expression data of 9736 tumors and 8587 normal samples from the TCGA and the GTEx projects, using a standard processing pipeline [8]. This tool was used to analyze the expression of ZWINT, including tumor/normal differential expression, profiling by cancer types or pathological stages, patient survival analysis, similar gene detection, correlation analysis and dimensionality reduction analysis.

### University of Alabama at Birmingham Cancer Data Analysis Portal (UALCAN)

In order to assess the dependability of the differential expression data, additional validation was conducted using the UALCAN (http://ualcan.path.uab.edu). In addition to evaluating gene expression in molecular subtypes of breast cancer and correlating other clinicopathological characteristics, UALCAN is designed to facilitate easy access to publicly available cancer OMICS data (TCGA, MET500, CPTAC and CBTTC), provide graphs and plots depicting pan-cancer gene expression and patient survival information based on gene expression, and evaluate epigenetic regulation of gene expression by promoter methylation. This tool was used to analyze the expression of ZWINT in pan-cancer and evaluate its prognostic significance through survival analysis.

### Differentially Expressed ZWINT at Protein Level

The ZWINT protein expression was systematically analyzed using information from the human protein atlas (HPA) (http://www.proteinatlas.org). Representative immunohistochemistry-based protein expression data for 20 extremely prevalent cancer types are available on the HPA platform. In our study, we specifically utilized HPA to examine and compare ZWINT protein expression patterns through direct visualization of immunohistochemical staining in both normal breast tissue and breast cancer specimens.

### Breast Cancer Gene-Expression Miner v5.1 (bc-GenExMiner v5.1)

By using the bc-GenExMiner v5.1 online dataset (http://bcgenex.centregauducheau.fr), the expression and prognostic value of ZWINT in breast cancer were evaluated. As a statistical mining tool, bc-GenExMiner v5.1 allows for gene expression, correlation, and prognosis analyses. The dataset, last updated on July 23, 2024, includes 5023 RNA-seq and 11,552 DNA microarray samples from patients. Using this tool, we assessed the correlation between ZWINT expression and clinicopathological characteristics of breast cancer.

### PrognoScan Online Database

We used the PrognoScan database (http://www.prognoscan.org/) to assess the prognostic significance of ZWINT mRNA expression in breast cancer patients. The analysis adjusted for significance by setting a corrected *P*-value threshold. The database automatically stratified patients into “high” and “low” ZWINT expression groups based on median expression levels, and generated Kaplan–Meier survival curves color-coded for clear visualization (red for high expression; blue for low expression).

### muTarget Analysis

The muTarget (https://www.mutarget.com/) integrates two complementary analytical modules designed to systematically investigate associations between target gene expression alterations and mutation profiles in human tumors. This methodology enables the identification of changes in target gene expression linked to specific gene mutations, as well as mutations that influence the expression of a chosen gene. All data processing was conducted using the R statistical software (version 4.0.3). RNA-seq and mutation data were sourced from the TCGA database, which contains 7876 solid tumor samples across 18 different tumor types, encompassing both RNA-seq data and somatic mutations. The effectiveness of this approach was demonstrated through three analyses in breast cancer: alterations in gene expression associated with CDH1 mutations, changes in gene expression related to TP53 mutations, and mutations that affect PGR expression. The breast cancer dataset was randomly stratified into two subsets for training and testing, with each set analyzed independently. Significant genes for the test were selected based on the Mann–Whitney *P*-value and mean fold change, utilizing the default thresholds of *P* ≤ 0.01, and FC < 0.714 or > 1.4.

### Construction of Related Gene Networks, GO, and KEGG Pathway Enrichment Analysis

To elucidate the functional context of ZWINT, we employed GeneMANIA (http://www.genemania.org), a web-based platform that generates functional association networks by identifying genes with similar biological roles to query genes. Our analysis revealed a comprehensive interaction network between ZWINT and its functionally associated partners. Subsequently, functional enrichment analysis was conducted through WebGestalt (http://www.webgestalt.org) and the Database for Annotation, Visualization, and Integrated Discovery (DAVID; https://davidbioinformatics.nih.gov/tools.jsp). DAVID was specifically employed to mitigate data redundancy while ensuring the retention of current functional annotations. Pathway analysis encompassing Kyoto Encyclopedia of Genes and Genomes (KEGG) pathways and Gene Ontology (GO) functional annotations was systematically performed on ZWINT and its 20 co-expressed genes using WebGestalt’s enrichment module. The method of interest was selected in Over-Representation Analysis (ORA). The GO functional enrichment was performed for the biological process (BP), cellular component (CC), and molecular function (MF). The pathway analysis was processed in the KEGG pathway.

### Clinical Bioinformatics Analysis Assistant

The clinical bioinformatics database assistant (http://www.aclbi.com) was utilized to examine the impact of genetic and clinicopathological characteristics, including age, sex, and TNM stages, on the prognosis of patients with breast cancer. Both univariate and multivariate Cox regression analyses were conducted to determine the appropriate variables for constructing the nomogram. Following the results of the multivariate Cox proportional hazards analysis, a nomogram was created to forecast the overall recurrence over X years. This nomogram offered a visual representation of the contributing factors, enabling the calculation of an individual patient’s recurrence risk based on the points assigned to each risk factor. All analytical procedures and R packages were executed using R software version v4.0.3, with a significance level set at *P* < 0.05.

## Results

### Overexpression of ZWINT mRNA in Breast Cancer Patients

**Fig. 1:**
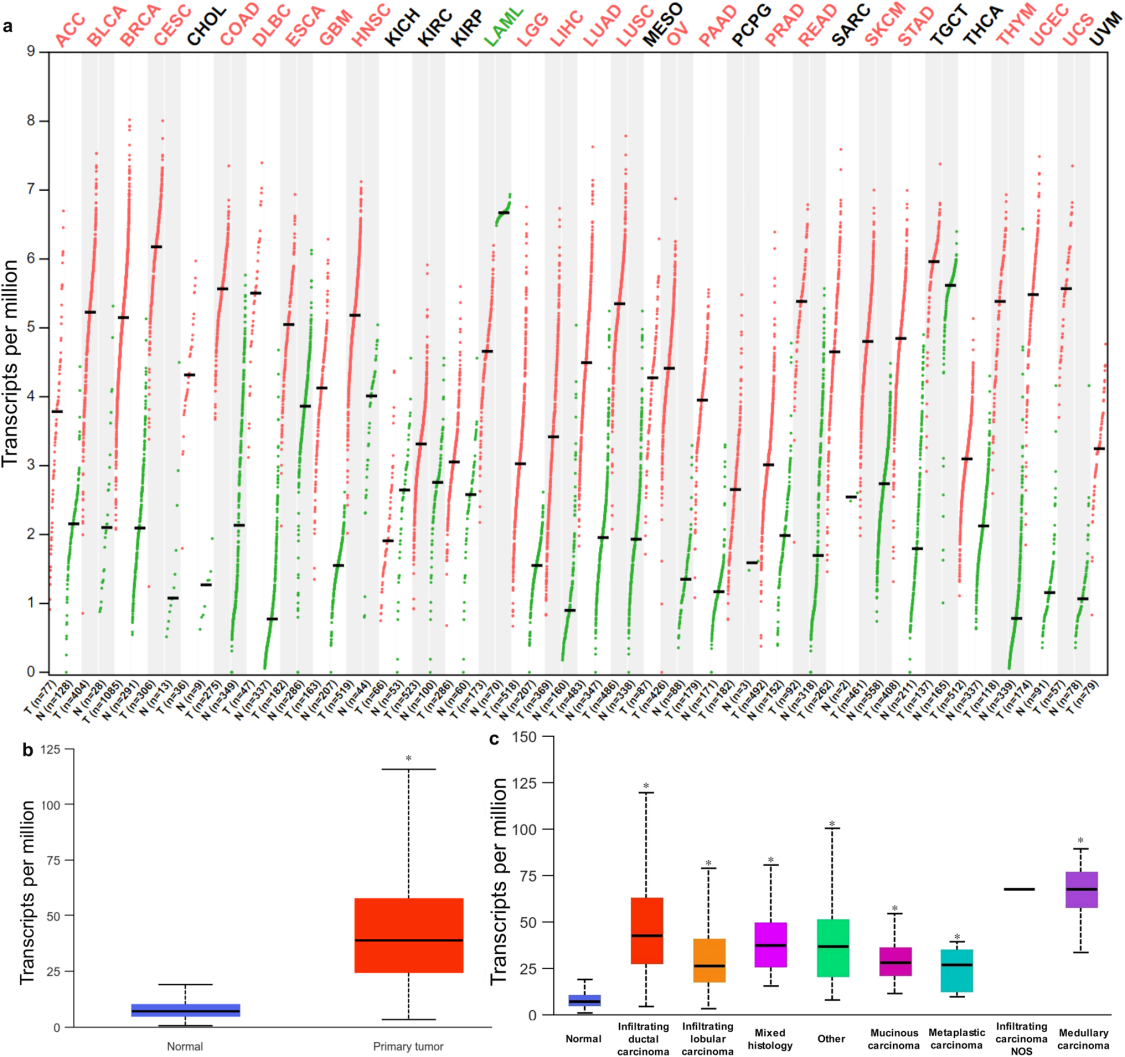
Detection of the ZWINT expression in 33 types of tumors and different types of breast cancer by using the TCGA database. **a** The pan-cancer analysis displayed the expression range of ZWINT across all tissues using RNA-seq data from normal and cancer tissues in the GEPIA database. **b** mRNA expression of ZWINT in patients with breast cancer and normal breast tissues by using UALCAN (TCGA database); **c** Boxplots comparing ZWINT expression between normal tissues and breast cancer subtypes using UALCAN (TCGA RNA-seq data) (^*^P < 0.05 vs. normal tissue). T, tumor tissue; N, normal tissue. ACC, adrenocortical carcinoma; BLCA, bladder urothelial carcinoma; BRCA, breast invasive carcinoma; CESC, cervical squamous cell carcinoma and endocervical adenocarcinoma; CHOL, cholangiocarcinoma; COAD, colon adenocarcinoma; DLBC, diffuse large B-cell lymphoma; ESCA, esophageal carcinoma; GBM, glioblastoma multiforme; HNSC, head and neck squamous cell carcinoma; KICH, kidney chromophobe; KIRC, kidney renal clear cell carcinoma; KIRP, kidney renal papillary cell carcinoma; LAML, acute myeloid leukemia; LGG, brain lower grade glioma; LIHC, liver hepatocellular carcinoma; LUAD, lung adenocarcinoma; LUSC, lung squamous cell carcinoma; MESO, mesothelioma; OV, ovarian serous cystadenocarcinoma; PAAD, pancreatic adenocarcinoma; PCPG, pheochromocytoma and paraganglioma; PRAD, prostate adenocarcinoma; READ, rectum adenocarcinoma; SARC, sarcoma; SKCM, skin cutaneous melanoma; STAD, stomach adenocarcinoma; TGCT, testicular germ cell tumors; THCA, thyroid carcinoma; THYM, thymoma; UCEC, uterine corpus endometrial carcinoma; UCS, uterine carcinosarcoma; UVM, uveal melanoma

Through comprehensive pan-cancer analysis utilizing the GEPIA, UALCAN, and TCGA databases, we systematically evaluated ZWINT expression profiles (Fig. 1). Initial analysis revealed significantly elevated ZWINT expression in breast cancer (median transcripts per million [TPM] = 5.15) compared to normal breast tissue (median TPM = 2.09) (Fig. 1a). This finding was further validated using UALCAN database analysis of TCGA RNA-seq data, which demonstrated marked upregulation of ZWINT in breast cancer tissues (Q1–Q3: 24.331–57.426; *n* = 1097) *versus* normal controls (Q1–Q3: 4.732–9.895; *n* = 114, *P* = 1.624E−12) (Fig. 1b). Notably, elevated ZWINT expression was consistently observed across all major breast cancer histological subtypes: infiltrating ductal carcinoma (Q1–Q3: 27.55–62.183, *n* = 784, *P* < 1.0E−12), infiltrating lobular carcinoma (Q1–Q3: 17.401–40.013, *n* = 203, *P* < 1.0E−12), infiltrating ductal and lobular carcinoma (Q1–Q3: 25.683–48.996, *n* = 29, *P* = 7.961E−11), mucinous carcinoma (Q1–Q3: 20.983–35.436, *n* = 17, *P* = 3.401E−03), medullary carcinoma (Q1–Q3: 57.865–76.304, *n* = 6, *P* = 8.365E−04) (Fig. 1c), compared with the normal tissue (Q1–Q3: 4.732–9.895, *n* = 114).

**Fig. 2:**
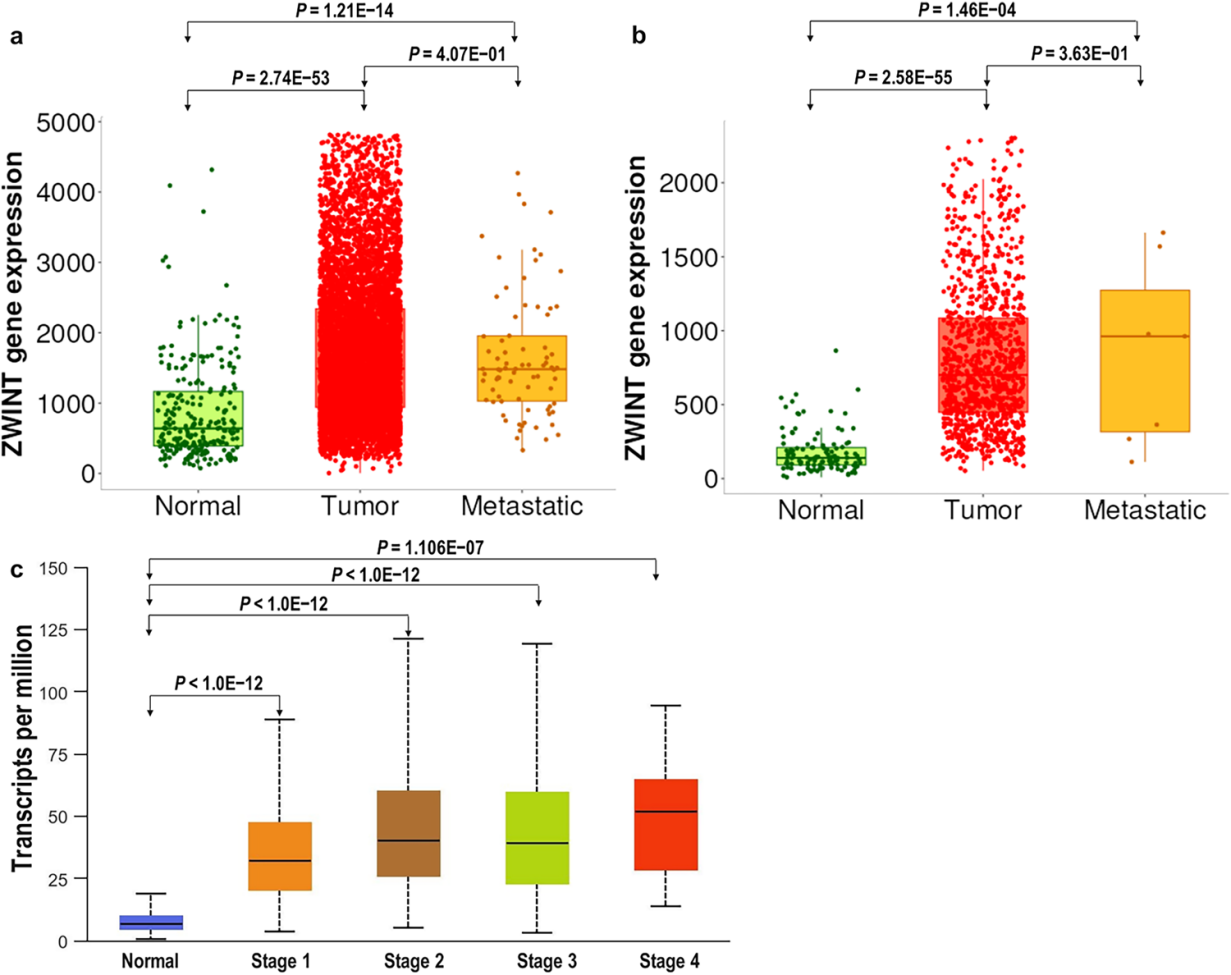
ZWINT expression in breast cancer tissues (normal, tumor, metastatic) by using gene chip & RNA-seq data from TNMplot database. **a** ZWINT expression from gene chip data; **b** ZWINT expression from RNA-seq data. Validation of differential expression using equally sized training and test sets confirmed the reliability of the database in breast cancer with an FDR below 10%. **c** Relationship between ZWINT expression and tumor stage in breast cancer patients. Boxplots displayed the ZWINT expression between normal and breast cancer patients with stage 1, 2, 3, and 4

Analysis of the TNMplot database revealed significant differential expression of ZWINT across breast cancer progression stages (Fig. 2). The results from gene chip data showed that ZWINT expression was higher in metastatic tissues (Q1–Q3: 967–2512, *n* = 7569) and tumor tissues (Q1–Q3: 1045–2346, *n* = 82) than in normal controls (Q1–Q3: 395–1200, *n* = 242) (*P* = 1.06E−51) (Fig. 2a). The results from RNA-seq data showed that ZWINT expression was also higher in metastatic tissues (Q1–Q3: 316.5–1273.5, *n* = 7) and tumor tissues (Q1–Q3: 467–1210, *n* = 1097) than in normal controls (Q1–Q3: 92–210, *n* = 112) (*P* = 1.01E−53) (Fig. 2b). Furthermore, ZWINT expression showed stage-dependent elevation in early stages (stage 1[Q1–Q3: 28.214–63.367, *n* = 97] vs. stage 2 [Q1–Q3: 25.039–61.347, *n* = 505], *P* < 0.05), but no significant difference was observed in advanced stages (stage 3 [Q1–Q3: 22.702–52.693, *n* = 431] vs. stage 4[Q1–Q3: 23.456–55.588, *n* = 54]). Notably, while advanced-stage tumors exhibited higher ZWINT expression compared to normal tissues (Q1–Q3: 4.732–9.895; *n* = 114), the expression levels did not show a progressive increase across different cancer stages (Fig. 2c). These findings collectively demonstrated that ZWINT mRNA was significantly upregulated during breast cancer development and progression.

### Overexpression of ZWINT in Breast Cancer Patients Based on HPA Database

We compared the ZWINT mRNA expression patterns between breast cancer samples from the HPA and normal breast tissues from the Genotype-Tissue Expression (GTEx) project, using RNA-seq data. Our analysis incorporated comprehensive sample characteristics, including gender, age, tissue section images, and estimated cell type fractions. Gene expression levels were quantified using normalized transcripts per million (nTPM), facilitating cross-sample comparisons.

**Fig. 3:**
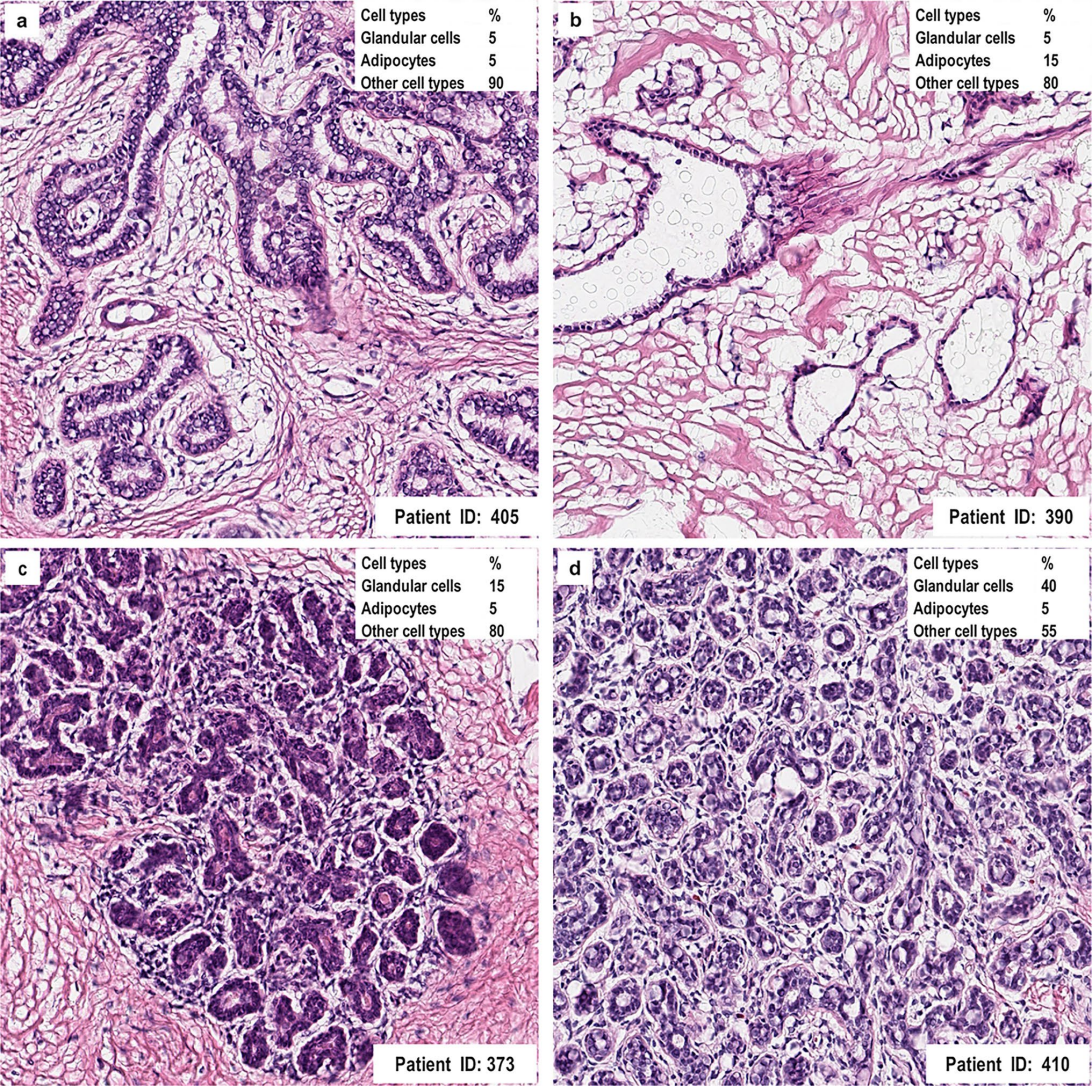
Analysis of ZWINT expression in the HPA database. **a** Patient ID 405 with 5% glandular cells; **b** Patient ID 390 with 5% glandular cells; **c** Patient ID 373 with 15% glandular cells; **d** Patient ID 410 with 40% glandular cells (magnification, ×400)

For instance, in breast sample No. 390, an 80-year-old patient, the nTPM value of ZWINT was 1.8 with 5% glandular cells (Fig. 3a). In sample No. 405, from a 47-year-old patient, the nTPM reached 4.4 with 5% glandular cells (Fig. 3b). Sample No. 373, from a 52-year-old patient, exhibited an nTPM of 5.2 and 15% glandular cells (Fig. 3c). Meanwhile, sample No. 410, obtained from a 38-year-old patient, showed a notably higher nTPM of 13.9 with 40% glandular cells (Fig. 3d).

Remarkably, the mean ZWINT expression in breast cancer tissues from HPA, with an average nTPM of 6.33 (*n* = 40), was significantly higher than that in normal breast tissues from GTEx (average nTPM = 1.42, *n* = 459). These results across multiple independent samples consistently indicate the upregulation of ZWINT in breast cancer patients.

### Correlation of ZWINT with Clinicopathologic Parameters in Breast Cancer Patients

**Fig. 4:**
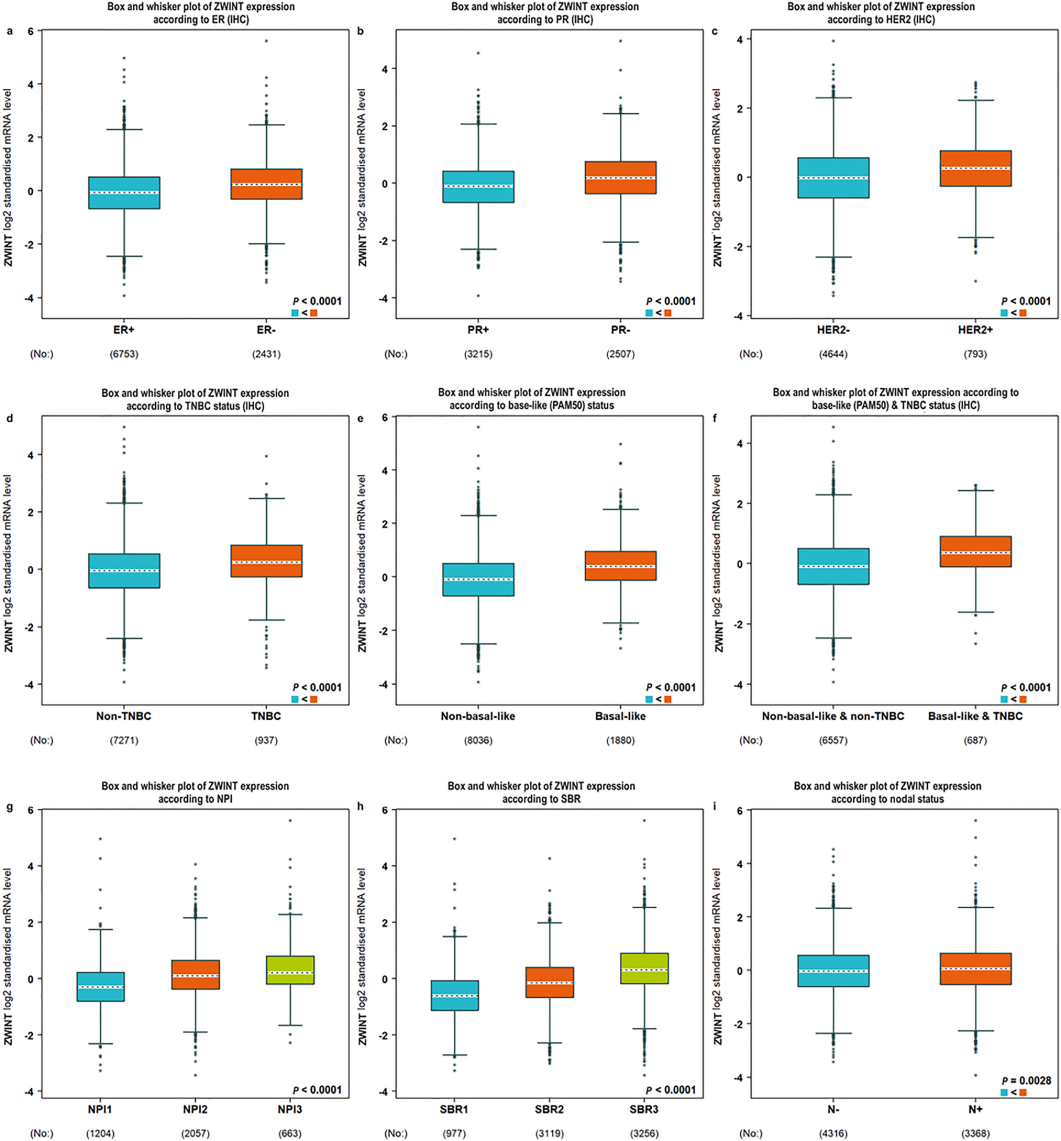
Correlation of ZWINT expression with clinicopathological parameters, including ER (**a**), PR (**b**), HER2 (**c**), TNBCs (**d**), basal-like cases (PAM50) (**e**), TNBCs and basal-like (PAM50) cases (**f**), NPI (**g**), SBR (**h**), and nodal status (**i**) in breast cancer patients. Notable differences between the groups were analyzed by Welch’s *t*-test to generate the *P* value. ZWINT, ZW10 interacting kinetochore protein; ER, estrogen receptor; PR, progesterone receptor; HER2, human epidermal growth factor receptor 2; SBR, Scarff, Bloom and Richardson; TNBC, triple-negative breast cancer; NPI, Nottingham prognostic index; IHC, immunohistochemistry

We systematically evaluated the association between ZWINT expression and clinicopathological parameters in breast cancer using bc-GenExMiner v5.1 (Fig. 4). All the expression levels of ZWINT mRNA are logarithmic values with a base of 2 (log₂(TPM)). To assess the significance of the difference in ZWINT gene distributions in between the different groups, a Welch’s test was performed, as well as Dunnett–Tukey–Kramer’s tests when appropriate. ZWINT mRNA expression was significantly downregulated in ER-positive patients (−0.0626 ± 0.9236, *n* = 6753) compared to ER-negative patients (0.2443 ± 0.9186, *n* = 2431) (*P* < 0.0001) (Fig. 4a). Similarly, ZWINT levels were markedly lower in PR-positive patients (−0.1158 ± 0.8860, *n* = 3215) than in PR-negative patients (0.1756 ± 0.8809, *n* = 2507) (*P* < 0.0001) (Fig. 4b). ZWINT mRNA expression was significantly lower in HER2-negative patients (−0.0147 ± 0.8913, *n* = 4644) compared to HER2-positive patients (0.2371 ± 0.8538, *n* = 793) (*P* < 0.0001) (Fig. 4c). Notably, analysis of TNBC patients revealed a distinct pattern, with ZWINT mRNA levels being markedly elevated in TNBC patients (0.2691 ± 0.8848, *n* = 937) relative to non-TNBC controls (−0.0452 ± 0.9204, *n* = 7271) (*P* < 0.0001) (Fig. 4d). Consistent with other findings, basal-like breast cancer patients demonstrated significantly elevated ZWINT expression levels (0.4245 ± 0.8502, *n* = 1880) compared to non-basal-like cases (−0.0868 ± 0.9233, *n* = 8036) (*P* < 0.0001) (Fig. 4e). Comparative analysis revealed significantly elevated ZWINT expression in both TNBC and basal-like subtype patients (0.4114 ± 0.7947, *n* = 687) compared to non-TNBC/non-basal-like patients (−0.0866 ± 0.9155, *n* = 6557) (*P* < 0.0001) (Fig. 4f). Analysis of the Nottingham Prognostic Index (NPI) revealed a significant positive correlation between ZWINT expression levels and increasing NPI grade (NPI1: −0.2782 ± 0.8260,* n* = 1204; NPI2: 0.1534 ± 0.8334, *n* = 2057; NPI3: 0.3093 ± 0.8374, *n* = 663) (*P* < 0.0001) (Fig. 4g). In the Scarff Bloom and Richardson grade (SBR) status, ZWINT expression showed progressive elevation with higher SBR grades (SBR1: −0.5944 ± 0.8649, *n* = 977; SBR2: −0.1251 ± 0.8292, *n* = 3119; SBR3: 0.3503 ± 0.8790, *n* = 3256) (*P* < 0.0001) (Fig. 4h). Analysis of nodal (N) status revealed significantly higher ZWINT expression in N (+) tumors (0.0598 ± 0.9086, *n* = 3368) compared to N (−) tumors (−0.0031 ± 0.9192, *n* = 4316) (*P* = 0.00028) (Fig. 4i).

### Correlation of ZWINT Expression with Crucial Genes Mutations

**Fig. 5:**
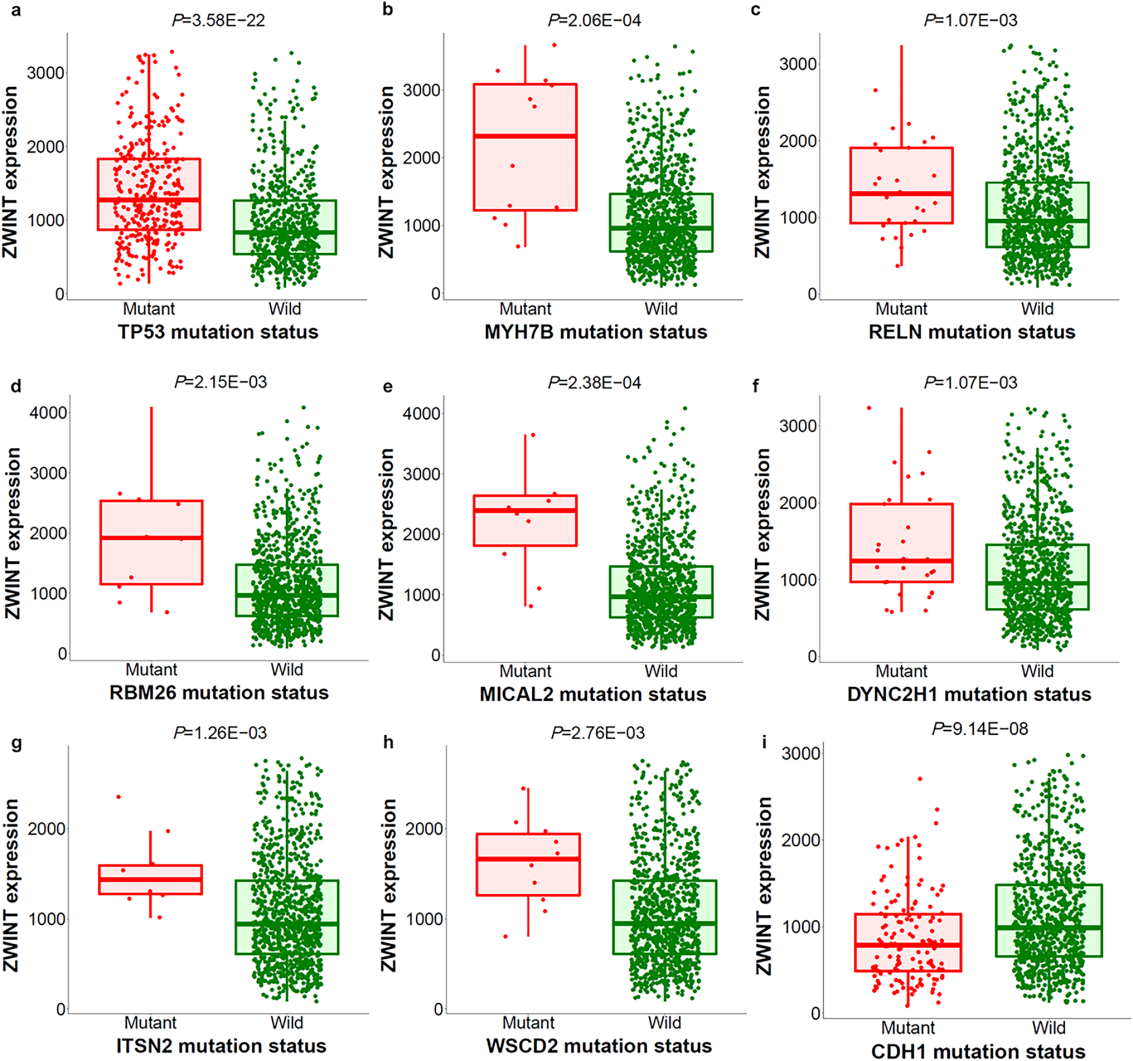
Identification of mutations correlated with ZWINT expression in breast cancer. The oncogenes or tumor suppressor genes mutations, which are correlated with ZWINT expression, were analyzed by TARGET from muTarget platform. The nine genes whose mutations are most strongly correlated with ZWINT expression changes in breast cancer, including TP53, tumor protein p53 (**a**); MYH7B, myosin heavy chain 7B (**b**); RELN, reelin (**c**); RBM26, RNA binding motif protein 26 (**d**); MICAL2, microtubule associated monooxygenase, calponin and LIM domain containing 2 (**e**); DYNC2H1, dynein cytoplasmic 2 heavy chain 1 (**f**); ITSN2, intersectin 2 (**g**); WSCD2, WSC domain containing 2 (**h**); CDH1, cadherin 1 (**i**). Screening of significant genes was based on the Mann–Whitney *P* value and mean FC of the test, with *P* ≤ 0.01 and 0.714 < FC < 1.4 and a prevalence of at least 1%

To investigate potential therapeutic implications, we performed Mann–Whitney analysis to identify significant somatic mutations associated with ZWINT expression levels in breast cancer. The selection criteria for genes were set as FC > 1.44 and *P* value < 0.01. There were 20 mutant genes strongly associated with ZWINT expression (Table S1). Upregulation of ZWINT was linked to 19 gene mutations, including TP53, MYH7B, and MICAL2, whereas downregulation of ZWINT was linked to a single gene mutation in CDH1. The nine genes most strongly correlated with ZWINT expression were identified (Fig. 5). Compared to wild-type cases, significantly higher ZWINT expression was observed in breast cancer patients harboring mutations in TP53 (total mutation rate: 34.321%) (Fig. 5a), MYH7B (total mutation rate: 1.328%) (Fig. 5b), RELN (total mutation rate: 3.269%) (Fig. 5c), RBM26 (total mutation rate: 1.124%) (Fig. 5d), MICAL2 (total mutation rate: 1.021%) (Fig. 5e), DYNC2H1 (total mutation rate: 3.269%) (Fig. 5f), ITSN2 (total mutation rate: 1.226%) (Fig. 5g), and WSCD2 (total mutation rate: 1.124%) (Fig. 5h). ZWINT expression was lower in tumor specimens from CDH1-mutant breast cancer patients (total mutation rate: 14.096%) (Fig. 5i). Collectively, these results suggested that ZWINT expression has a close correlation with gene mutations in breast cancer.

### Construction Gene Interaction Network and Function Enrichment of ZWINT in Breast Cancer

**Fig. 6:**
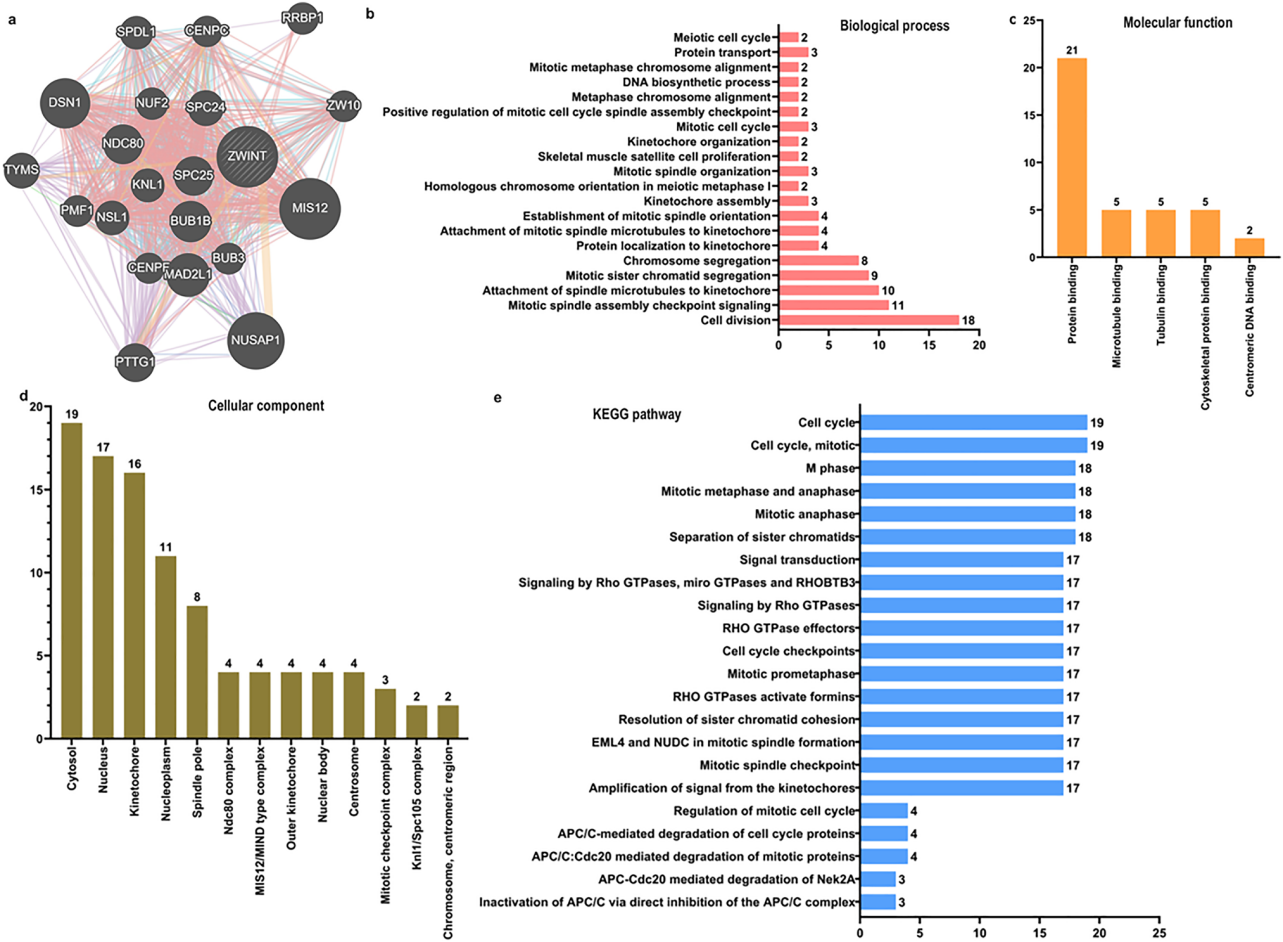
Protein–protein interaction network and function enrichment analysis of ZWINT in breast cancer. **a** The protein–protein interaction network of ZWINT was analyzed using GeneMANIA, and 20 genes that interact with ZWINT were screened. The different colors of the network edges represent various bioinformatics methods employed, including Physical Interactions, Co-expression, Predicted, Co-localization, Pathway, Genetic Interactions, and Shared Protein Domains. **b** Gene Ontology (GO) analysis was conducted, and the 20 screened genes related to biological process (BP) are presented. **c** Results for Cellular Component (CC) are shown. **d** Results for molecular function (MF) are shown. **e** KEGG pathway analysis was performed on the 20 genes co-expressed with ZWINT. The top pathways were mapped based on the differential expression level of ZWINT (*P* ≤ 0.05). The Y-axis represents the names of the signaling pathways or functions, while the X- axis indicates the percentage of the number of genes assigned to a specific term relative to the total number of genes annotated in the network

We established a protein–protein interaction network for ZWINT and its 20 associated genes using GeneMANIA (Fig. 6). Proteins interacting with ZWINT, identified via GeneMANIA, were MIS12, NUSAP1, DSN1, MAD2L1, BUB1B, NDC80, SPC25, PTTG1, TYMS, SPC24, NSL1, KNL1, SPDL1, NUF2, RRBP1, BUB3, ZW10, PMF1, CENPC, and CENPF (Fig. 6a). Furthermore, we conducted functional enrichment analysis of the 20 genes using WebGestalt. The results demonstrated that these genes were significantly enriched in three major categories: biological processes (Fig. 6b), molecular functions (Fig. 6c), and cellular components (Fig. 6d). Notably, most of the genes were significantly enriched in metabolic processes, biological regulation, and protein binding, among other functions. Furthermore, GO and pathway analysis demonstrated that these proteins exhibited the strongest association with the cell cycle. Functional enrichment analysis revealed that these genes were significantly associated with sister chromatid separation, RHO GTPase-mediated formation activation, kinetochore signal amplification, and carbon metabolism pathways (Fig. 6e).

### ZWINT as an Independent Indicator for Breast Cancer Prognosis

**Fig. 7:**
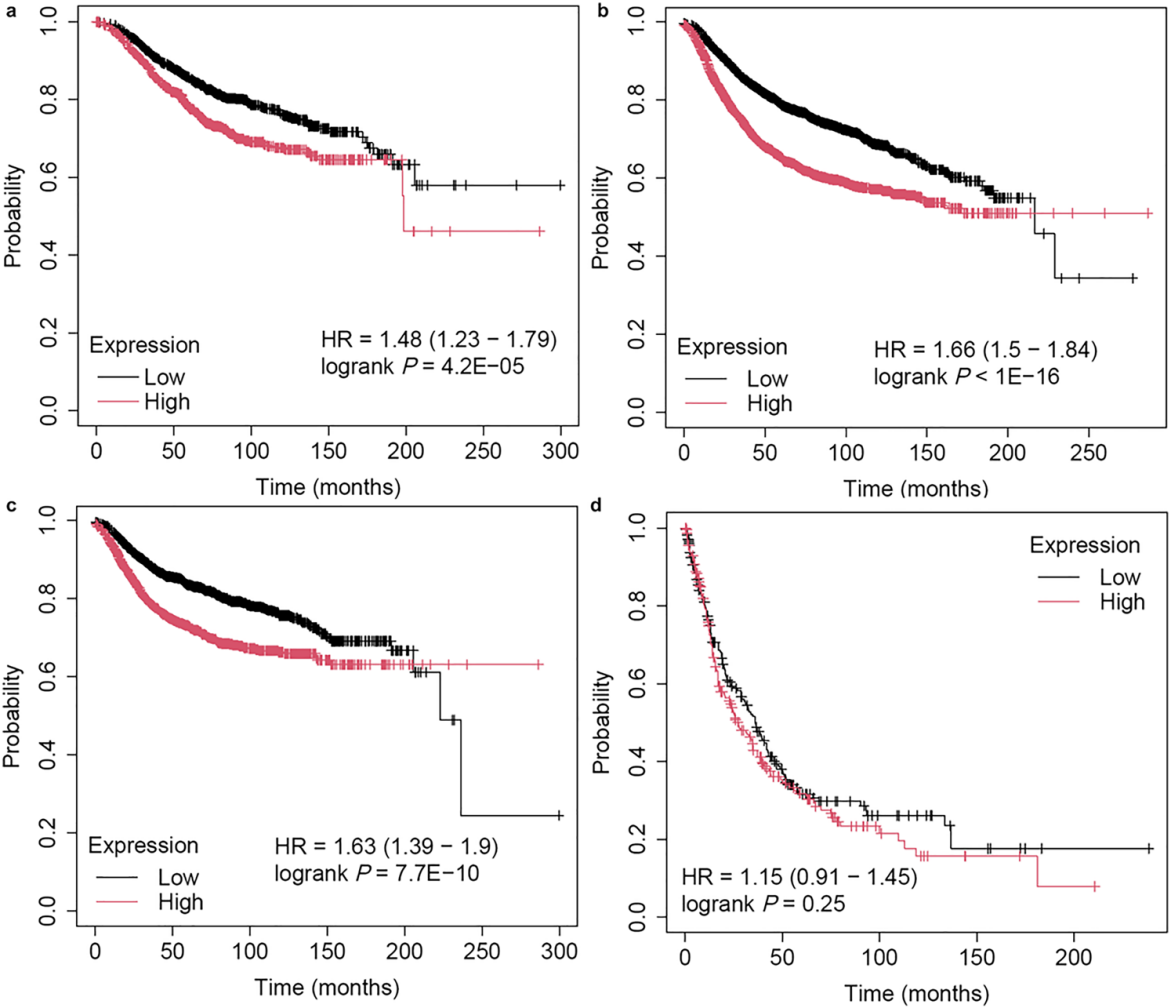
Prognostic significance of ZWINT expression in patients with breast cancer. **a** The relationship between ZWINT expression and OS time; **b** the relationship between ZWINT expression and RFS time; **c** the relationship between ZWINT expression and DMFS time; **d** the relationship between ZWINT expression and PPS time. OS, overall survival; RFS, relapse-free survival; DMFS, distant metastasis free survival; HR, hazard ratio; 95% CI, 95% confidence interval

To evaluate the prognostic significance of ZWINT expression in breast cancer, we analyzed its correlation with patient survival outcomes, including overall survival (OS), relapse-free survival (RFS), distant metastasis-free survival (DMFS), and progress-free survival (PPS) (Fig. 7). First, we analyzed the relationship between ZWINT expression and OS, RFS, DMFS, or PPS across all factors. For OS, the upper quartile survival time of patients with high ZWINT expression was 68.4 months, compared with 130 months for those with low expression, indicating a shorter OS in the high-expression cohort (hazard ratio [HR] 1.48, 95% confidence interval [CI] 1.23–1.79, *P* = 4.2E−05) (Fig. 7a). In terms of RFS, the upper quartile survival time was 35 months for high-ZWINT-expressing patients and 80.4 months for low-expressing patients, suggesting a shorter RFS for the former group (HR 1.66, 95% CI 1.5–1.84, *P* < 1E−16) (Fig. 7b). Regarding DMFS, the upper quartile survival time of high-ZWINT- expressing patients was 48 months, while that of low-expressing patients was 130.72 months, implying a shorter DMFS in the high-expression cohort (HR 1.63, 95% CI 1.39–1.9, *P* = 7.7E−10) (Fig. 7c). For PPS, the median survival time was 27.6 months in high-ZWINT-expressing patients and 36 months in low-expressing patients, indicating no significant difference (HR 1.15, 95% CI 0.91–1.45, *P* = 0.2458) (Fig. 7d).

Subsequently, we analyzed the impact of ZWINT expression on patient prognosis under different clinicopathologic characteristics, including ER status, PR status, HER2 status, lymph node status, TP53 status, clinical grade, and molecular subtype-PAM50 (Table S2). Among ER-positive patients, the upper quartile survival time was 75.83 months for those with high ZWINT expression and 175.46 months for low-expression patients, suggesting a shorter OS in the high-expression group (HR 1.87, 95% CI 1.36–2.58, *P* = 9.2E−5). High ZWINT expression was also associated with shorter RFS (HR 1.74, 95% CI 1.49–2.03, *P* = 1.1E−12) and DMFS (HR 1.95, 95% CI 1.47–2.57, *P* = 1.8E−6) in ER-positive patients. In contrast, among ER-negative patients, high ZWINT expression was linked to shorter OS (HR 1.42, 95% CI 1.14–1.78, *P* = 0.0016), RFS (HR 1.63, 95% CI 1.45–1.83, *P* < 1E−16), and DMFS (HR 1.57, 95% CI 1.31–1.87, *P* = 4.5E−7). Additionally, high ZWINT expression was associated with shorter OS (HR 1.97, 95% CI 1.39–2.8, *P* = 0.0001), RFS (HR 1.46, 95% CI 1.24–1.72, *P* = 3.7E−6), and DMFS (HR 1.71, 95% CI 1.33–2.19, *P* = 2.0E−5) in another analysis of ER-negative patients. However, high ZWINT expression was not significantly associated with OS time in patients with TP53 mutation or across different PAM50 molecular subtypes (*P* ≥ 0.05).

**Fig. 8:**
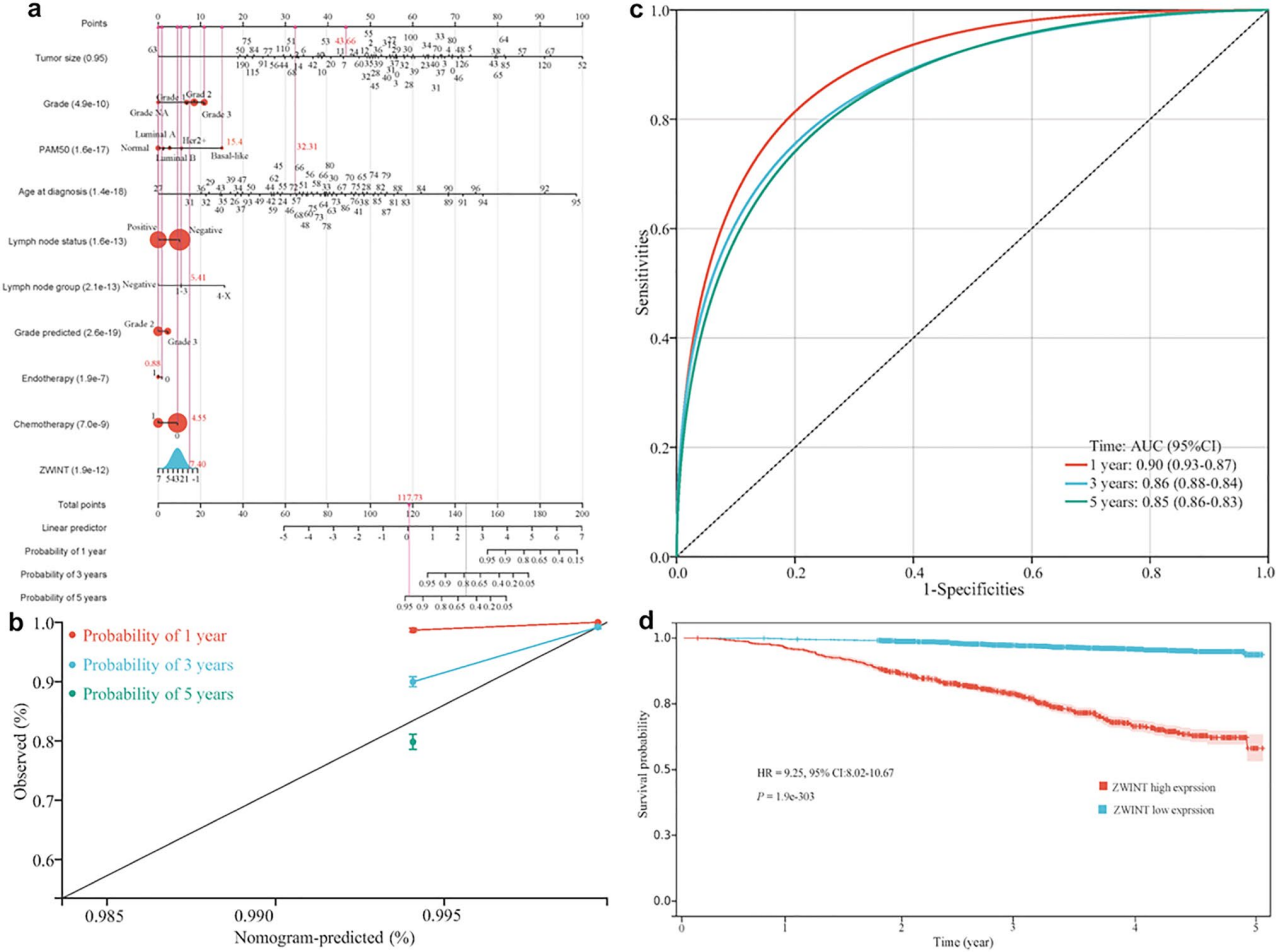
Multivariate regression model to predict the risk of overall survival of patients with breast cancer. **a** Calibration curve for the overall survival nomogram model in the breast cancer cohort. The dashed diagonal line symbolizes the ideal nomogram, while the red, orange, and blue lines represent the observed nomograms for 1, 3, and 5-year survival, respectively. **b** nomogram to predict the 1, 3, and 5-year overall survival of breast cancer patients; **c** time-dependent ROC analysis of the 5-gene signature; **d** prognostic significance of ZWINT expression in breast cancer patients with respect to overall survival. ROC, receiver operating characteristic

Additionally, we used the R rms package to integrate data on survival time, survival status, and 10 features (Fig. 8). Using the Cox method, we established a nomogram and evaluated the prognostic significance of these features in 3374 samples. The overall Harrell’s C-index of the model was 0.7827, with 95% CI 0.7575–0.8079, *P* = 2.1627E−107 (Fig. 8a). Calibration analysis demonstrated that the nomogram achieved optimal predictive accuracy for 1-year survival (red line), closely approximating the ideal 45-degree reference line (gray). While maintaining good concordance for 3-year (orange line) and 5-year (blue line) survival predictions, the model showed particularly remarkable accuracy for short-term (1-year) prognosis (Fig. 8b). Receiver operating characteristic (ROC) analysis was performed using the pROC package (v1.17.01) in R. We evaluated the model’s predictive performance at 1-, 3-, and 5-year time points using the package’s ROC function, with area under the curve (AUC) values and corresponding 95% confidence intervals calculated via the ci function. The analysis demonstrated excellent discriminative ability, with AUC values of 0.90 (95% CI: 0.87–0.93) for 1-year, 0.86 (95% CI: 0.84–0.88) for 3-year, and 0.85 (95% CI: 0.83–0.86) for 5-year predictions (Fig. 8c). The optimal cutoff value for the risk score was determined using the maxstat package (version 0.7–25) in R, which implements maximally selected rank statistics with multiple *P*-value approximation methods. We constrained the analysis by requiring each risk group to comprise between 25% and 75% of the total sample population. This approach yielded an optimal risk score cutoff value of 0.685 (Fig. 8c). Based on this, patients were stratified into high-risk and low-risk groups. Subsequent OS analysis was performed using the survival package in R to compare prognostic outcomes between these groups. The likelihood ratio test was employed to assess the statistical significance of survival differences, revealing an extremely significant divergence in prognosis between the risk groups (*P* = 1.9E−303) (Fig. 8d).

## Discussion

With the rapid advancement of precision medicine, accurate prognostic models have become essential for guiding clinical care and tailoring personalized treatment programs, especially for patients with complex breast cancer subtypes. Breast cancer is a life-threatening disease characterized by a high metastatic potential [9]. Among the various molecular players involved in breast cancer progression, ZWINT has emerged as a key regulator.

During mitotic prometaphase, ZWINT is intricately involved in kinetochore function. Beyond its role in cell division, mounting evidence indicates that ZWINT is critical for tumor cell invasion and proliferation. Aberrant ZWINT expression has been detected in diverse tumor tissues, as well as in patient peripheral blood and saliva samples [10]. Such dysregulation may contribute to accelerated tumor growth and progression, making ZWINT an attractive therapeutic target [5].

Notably, studies have shown a strong correlation between elevated ZWINT expression and advanced tumor staging, suggesting its involvement in breast cancer metastasis and disease progression [11]. However, the expression profiles of ZWINT across different breast cancer subtypes have not been comprehensively characterized. Additionally, the specific molecular mechanisms underlying ZWINT’s role as a biomarker for poor prognosis require further investigation. Addressing these questions will be crucial for fully harnessing the potential of ZWINT in breast cancer management.

ZWINT expression was higher in breast cancer tissues than in healthy breast tissues according to the earlier research [12]. The ZWINT mRNA expression was also significantly upregulated in invasive ductal breast carcinoma, intraductal cribriform breast adenocarcinoma, invasive ductal and lobular carcinoma, mixed lobular and ductal breast carcinoma, invasive breast carcinoma, and invasive lobular breast carcinoma, according to the results of our comprehensive analysis of the Oncomine database, which we synthesized and analyzed based on the existing basic scientific research [11]. The analysis of data from the TNMplot and UALCAN databases revealed a significant correlation between ZWINT transcription levels and both metastasis and cancer stages. Additionally, HPA indicated that ZWINT protein expression was elevated in breast cancer tissues compared to normal tissues. Furthermore, the bc-GenExMiner 5.1 tool was utilized to explore the expression profile of ZWINT across various PAM50 breast cancer subtypes, taking into account different clinicopathological parameters. Elevated ZWINT expression was linked to an increased risk of HER2 positivity, TNBC, basal-like traits, SBR grade status, and NPI grade status. Conversely, ZWINT mRNA expression was notably reduced in patients with ER-positive and PR-positive statuses.

Cyclin-dependent kinase 1 (CDK1) serves as a catalytic subunit of the highly conserved protein kinase complex known as the M-phase promoting factor (MPF), which is essential for the G2/M phase transitions of the eukaryotic cell cycle. Moreover, ZWINT is directly regulated by CDK1 in many cancer types [13, 14], and related to cancer cell metastatic ability through CDK1/ZWINT-mediated epithelial-mesenchymal transition (EMT) process [15]. In our study, GeneMANIA and WebGestalt analysis indicated ZWINT was involved in mitotic prometaphase, separation of sister chromatids, cell division, and CDK1-signaling pathway. This result is consistent with previous studies [12] and also indicates that ZWINT plays an energetical role in tumor cell growth, invasion, and migration. In the future, further investigation is needed to clarify the signaling pathways through which ZWINT mediates tumor growth, invasion, and migration.

Precise prognostic models can assist in informing clinical decision-making and creating more tailored treatment strategies for patients [16]. The analyzed data indicate that high ZWINT expression is associated with reduced OS across various cancer types [17], and ZWINT alongside other genes can enhance predictions regarding the survival of breast cancer patients. Concerning the constrained effectiveness of current prognostic models for breast cancer, we conducted a thorough analysis of the prognostic significance of ZWINT alongside clinical variables such as age, race, and pTNM stage. The survival analysis indicated that elevated levels of ZWINT mRNA correlated with OS, RFS, and DMFS, as determined by PrognoScan analysis. Furthermore, ZWINT emerged as an independent prognostic factor for breast cancer through both univariate and multivariate Cox regression analyses. The breast cancer prognostic model based on ZWINT demonstrated a moderate predictive capability, with area under the curve (AUC) values of 0.90, 0.86, and 0.85 for 1-, 3-, and 5-year survival rates, respectively. Consequently, the ZWINT biomarker stands out as an independent and critical risk factor for breast cancer prognosis.

In recent years, multigene prognostic models have gained traction alongside single-gene indicators [18]. However, despite these advancements, many breast cancer patients with various somatic mutations still lack sufficient targeted treatment options. This highlights the need to explore more genes and their interactions to develop effective personalized therapies. To fill this gap, we employed the MuTarget software to identify mutations that impact the expression of ZWINT.

The TP53 gene, encoding a tumor suppressor protein, plays a crucial role in prognosis, especially in many cancer patients with TP53 mutations [19, 20]. Notably, breast cancer accounts for more than 50% of tumors in individuals carrying TP53 mutations [21, 22]. Our MuTarget analysis revealed that the expression of ZWINT was elevated in TP53-mutant breast cancer patients compared to those with wild-type TP53. Under normal circumstances, TP53 may act on NUSAP1 indirectly or directly through MDM2/4 to regulate the expression of ZWINT. When TP53 mutates, this regulatory effect is weakened, resulting in the upregulation of the ZWINT expression.

In addition, our present analysis indicated that ZWINT expression levels were reduced in CDH1-mutant breast cancer patients compared to those with the wild-type. CDH1 is frequently mutated in breast cancer cases [23]. This gene encodes a tumor suppressor protein, and its mutations play a significant role in promoting the invasion and metastasis of breast cancer [24]. CDH1 may indirectly regulate the expression of ZWINT through CAPNS1 by acting on NUSAP1. When CDH1 mutates, this regulatory effect is weakened, leading to the downregulation of the ZWINT expression. By correlating TP53 and CDH1 mutations with ZWINT expression, the potential multi-gene therapeutic targets can be identified, which provides a basis for the development of innovative personalized treatments for breast cancer.

## Conclusion

In summary, our data analysis reveals that ZWINT is overexpressed at both the transcriptional and protein levels in patients with breast cancer. ZWINT shows a significant correlation with various molecular subtypes, metastasis status, and the pTNM stage of patients. ZWINT overexpression is notably associated with reduced OS, RFS, and DMFS in breast cancer patients, and ZWINT has the potential to serve as an independent prognostic marker in the patients. Our data also suggest ZWINT may represent a promising target for the development of anti-ZWINT therapies in addition to being an effective prognostic indicator for breast cancer. This study was conducted using experimental data sourced from public databases. Consequently, further experimental validation of the mechanisms by which ZWINT affects tumor metastasis and its multi-gene prognostic significance in breast cancer patients is essential.

## Supplementary Information

Below is the link to the electronic supplementary material.

Supplementary file1 (DOCX 29 KB)

## Data Availability

The datasets presented in this study can be found in online repositories. The names of the repository/repositories and accession number(s) can be found in the article.
