# Supplementary material for: Association of ZWINT Expression with Clinicopathologic Characteristics and Prognosis in Breast Cancer Patients
Source: Curr Med Sci. 2025 Jul 1;45(4):775–88. doi: 10.1007/s11596-025-00081-9 (PMC12364763; doi:10.1007/s11596-025-00081-9)
Supplement: Supplementary file 1 — Supplementary file1 (DOCX 29 KB) [file 11596_2025_81_MOESM1_ESM.docx]

**Table S1 The twenty mutant genes is strongly associated with ZWINT expression**

| Mutation of gene | Mean expression (mutant) | Mean expression (wild) | Number of mutation | Number of wild | FC (mutant/wild) | Direction | *P*-value |
| --- | --- | --- | --- | --- | --- | --- | --- |
| TP53 | 1506.6 | 1030.67 | 336 | 643 | 1.46 | Up | 3.58E-22 |
| MYH7B | 2721.46 | 1173.46 | 13 | 966 | 2.32 | Up | 2.06E-04 |
| MICAL2 | 2357.6 | 1182.01 | 10 | 969 | 1.99 | Up | 2.38E-04 |
| RELN | 1766.19 | 1174.68 | 32 | 947 | 1.5 | Up | 1.07E-03 |
| DYNC2H1 | 1729.16 | 1175.93 | 32 | 947 | 1.47 | Up | 1.07E-03 |
| ITSN2 | 1975.08 | 1184.32 | 12 | 967 | 1.67 | Up | 1.26E-03 |
| RBM26 | 2277.09 | 1181.71 | 11 | 968 | 1.93 | Up | 2.15E-03 |
| WSCD2 | 1847 | 1186.59 | 11 | 968 | 1.56 | Up | 2.76E-03 |
| TMEM131 | 1881.08 | 1184.77 | 13 | 966 | 1.59 | Up | 3.59E-03 |
| COL1A1 | 1971.58 | 1184.37 | 12 | 967 | 1.66 | Up | 3.71E-03 |
| NCKAP5 | 1776.75 | 1184.33 | 16 | 963 | 1.5 | Up | 4.97E-03 |
| MIA3 | 1952.95 | 1178.99 | 19 | 960 | 1.66 | Up | 4.98E-03 |
| OGDHL | 2035.73 | 1184.45 | 11 | 968 | 1.72 | Up | 4.99E-03 |
| UBA6 | 1986.18 | 1185.01 | 11 | 968 | 1.68 | Up | 5.94E-03 |
| KCNA1 | 1849.6 | 1187.25 | 10 | 969 | 1.56 | Up | 6.15E-03 |
| UNC5D | 1768.88 | 1183.86 | 17 | 962 | 1.49 | Up | 6.63E-03 |
| FGD6 | 2631.3 | 1179.18 | 10 | 969 | 2.23 | Up | 6.99E-03 |
| CTNND2 | 2537 | 1177.35 | 12 | 967 | 2.15 | Up | 7.01E-03 |
| SLC4A2 | 1913.09 | 1185.84 | 11 | 968 | 1.61 | Up | 7.38E-03 |
| CDH1 | 862.71 | 1248.38 | 138 | 841 | 1.45 | Down | 9.14E-08 |

**Table S2 The impact of ZWINT gene expression on patient prognosis under different clinicopathologic characteristics**

| Index | OS | | | | RFS | | | | DMFS | | | | PPS | | | |
| --- | --- | --- | --- | --- | --- | --- | --- | --- | --- | --- | --- | --- | --- | --- | --- | --- |
|  | **Survival time (months)** | | **HR (95% CI)** | ***P*** | **Survival time (months)** | | **HR (95% CI)** | ***P*** | **Survival time (months)** | | **HR (95% CI)** | ***P*** | **Survival time (months)** | | **HR (95% CI)** | ***P*** |
|  | **Low (*****n*)** | **High (*n*)** |  |  | **Low (*n*)** | **High (*n*)** |  |  | **Low (No.)** | **High (*n*)** |  |  | **Low (*n*)** | **High ( *n*)** |  |  |
| ER status | | | | | | | | | | | | | | | | |
| Negative | 59.76 (260) | 72.2 (260) | 0.83 (0.59-1.15) | 0.26 | *136.87 (595) | *171.43 (595) | 1 (0.83-1.21) | 1 | 32.78 (374) | 29.9 (373) | 0.96 (0.74-1.24) | 0.75 | *42.48 (58) | *31.8 (58) | 1.14 (0.7-4.87) | 0.6 |
| Positive | 175.46 (377) | 75.83 (377) | 1.87 (1.36-2.58) | 9.2e-5 | 111.1 (1316) | 51 (1317) | 1.74 (1.49-2.03) | 1.1e-12 | *236.22 (570) | *205.64 (571) | 1.95 (1.47-2.57) | 1.8e-6 | *48 (100) | *34.68 (101) | 1.25 (0.87-1.81) | 0.225 |
| PR status | | | | | | | | | | | | | | | | |
| Negative | (146) | (145) | 0.98 (0.6-1.6) | 0.9461 | 35.42 (462) | 32.03(463) | 1.01 (0.8-1.27) | 0.9455 | 33.96 (318) | 30.17 (319) | 1.02 (0.76-1.36) | 0.912 | *39.24 (18) | *56.16 (19) | 1.14 (0.42-3.09) | 0.789 |
| Positive | (78) | (78) | 1.44 (0.68-3.05) | 0.3387 | 108 (463) | 61(463) | 1.49(1.11-1.99) | 0.007 | (264) | (265) | 1.63 (1.02-2.59) | 0.038 | 20.64 (16) | 50.16 (16) | 0.44 (0.15-1.3) | 0.129 |
| HER2 status | | | | | | | | | | | | | | | | |
| Negative | 136.8 (730) | 84 (729) | 1.42 (1.14-1.78) | 0.0016 | 86.9 (2024) | 37.51(2023) | 1.63(1.45-1.83) | <1e-16 | 133.15 (1157) | 61.2 (1157) | 1.57 (1.31-1.87) | 4.5e-7 | 36.99 (174) | *34.68 (173) | *1.08 (0.82-1.41) | 0.593 |
| Positive | 62.83 (210) | 56.64 (210) | 1.2 (0.84-1.73) | 0.3161 | 162.58 (441) | *161.29 (441) | 1.26(1.02-1.57) | 0.0356 | 36.56 (226) | 38.4 (225) | 0.99 (0.71-1.38) | 0.941 | 25.48 (56) | *18 (55) | *1.04 (0.66-1.63) | 0.879 |
| Lymph node status | | | | | | | | | | | | | | | | |
| Negative | 191.21 (363) | 89.03 (363) | 1.97 (1.39-2.8) | 0.0001 | 116 (1185) | 60.12 (1183) | 1.46 (1.24-1.72) | 3.7e-6 | 143.11 (654) | 78.02 (655) | 1.71 (1.33-2.19) | 2.0e-5 | *58.19 (92) | *52.7 (92) | 1.26 (0.84-1.9) | 0.260 |
| Positive | 50 (227) | 45.24 (225) | 1.14(0.82-1.58) | 0.4448 | 50 (828) | 30.72 (828) | 1.48 (1.25-1.76) | 4.7e-6 | 53 (444) | 30.98 (445) | 1.36 (1.06-1.75) | 0.015 | *26.8 (76) | *24.96 (77) | 1.19 (0.79-1.79) | 0.399 |
| TP53 status | | | | | | | | | | | | | | | | |
| Wild | (98) | (99) | 2 (1.06-3.77) | 0.0302 | 107.05 (137) | 47 (136) | 1.51 (0.99-2.31) | 0.0558 | (60) | (59) | 2.88 (1.32-6.31) | 0.0055 | 57.96 (31) | *62.99 (31) | *1.26 (0.64-2.51) | 0.505 |
| Mutated | 56 (65) | 66 (65) | 1.06 (0.55-2.07) | 0.8557 | 29.04 (94) | 21.96 (94) | 1.1 (0.69-1.77) | 0.689 | 24.07 (52) | 56(51) | 0.66(0.31-1.41) | 0.2801 | *32.96 (27) | *18 (27) | 1.19 (0.5-2.83) | 0.687 |
| Grade | | | | | | | | | | | | | | | | |
| 1 | *191.21(88) | *197.62(87) | 1.58 (0.66-3.83) | 0.303 | 160.08 (199) | 114.02 (198) | 1.84 (1.09-3.09) | 0.0206 | 191.21 (120) | 137 (119) | 2.59 (1.11-6.09) | 0.0241 | *68.96 (18) | *109.71 (17) | 0.93 (0.34-2.53) | 0.885 |
| 2 | 175.46 (222) | 80.65(221) | 1.62 (1.08-2.42) | 0.0186 | *216.66 (588) | *162.58 (589) | 1.55 (1.24-1.93) | 7.9e-5 | 130.72 (399) | 63.06 (399) | 1.56 (1.16-2.1) | 0.0028 | *48 (71) | *39.25 (71) | 1.19 (0.75-1.87) | 0.467 |
| 3 | *205.64 (293) | *198.44 (293) | 0.99 (0.73-1.34) | 0.9548 | 35.98 (650) | 28.35 (650) | 1.1 (0.91-1.33) | 0.3094 | 41.92 (418) | 37 (418) | 1.05 (0.81-1.36) | 0.711 | *32.47 (94) | 24.96 (93) | 0.99 (0.69-1.41) | 0.958 |
| Molecular subtype - PAM50 | | | | | | | | | | | | | | | | |
| Basal-like | 63 (216) | 115 (215) | 0.7 (0.47-1.02) | 0.0639 | 28 (476) | 21 (477) | 1.04 (0.84-1.28) | 0.7432 | 34.46 (315) | 31.33 (315) | 0.9 (0.67-1.2) | 0.4635 | *19.4 (38) | *33.7 (39) | 0.8 (0.46-1.39) | 0.424 |
| Luminal A | 175.46 (298) | 173.72 (298) | 1.19 (0.78-1.82) | 0.4296 | *216.66 (905) | *228.85 (904) | 1.4 (1.14-1.72) | 0.0015 | *236.22 (500) | *222.81 (498) | 1.32 (0.95-1.83) | 0.0925 | *65.95 (64) | 28.8 (64)* | 1.83 (1.12-2.98) | 0.014 |
| Luminal B | *197.62 (220) | *198.44 (219) | 1.01 (0.71-1.43) | 0.976 | 47 (677) | 35.09 (676) | 1.19 (1-1.42) | 0.0484 | 66.31 (336) | 47.52 (337) | 1.15 (0.86-1.55) | 0.3389 | *36 (74) | *34.68 (74) | 1.06 (0.71-1.58) | 0.777 |
| HER2+ | 50.67 (181) | 51.6 (181) | 1.05 (0.71-1.54) | 0.8153 | *162.58 (348) | *171.43 (347) | 0.97 (0.77-1.24) | 0.8219 | 29.9 (200) | 38.31 (201) | 0.84 (0.59-1.2) | 0.3365 | *20.04 (46) | *16.8 (46) | 1.06 (0.65-1.73) | 0.804 |
| Normal | 78 (26) | 27 (25) | 2.02(0.73-5.56) | 0.1653 | 70.8 (60) | 35 (59) | 1.42 (0.72-2.79) | 0.3094 | (32) | (31) | 3.42 (1.2-9.77) | 0.0147 | NA | NA | NA | NA |

*Means median survival time, other means upper quartile survival time.
